# The Role of Perceived In-group Moral Superiority in Reparative Intentions and Approach Motivation

**DOI:** 10.3389/fpsyg.2017.00912

**Published:** 2017-05-31

**Authors:** Zsolt P. Szabó, Noémi Z. Mészáros, István Csertő

**Affiliations:** ^1^Department of Social Psychology, Eötvös Loránd University Budapest, Hungary; ^2^Department of Social and Organizational Psychology, University of Pécs Pécs, Hungary; ^3^Department of Social and Organizational Psychology, Pázmány Péter Catholic University Budapest, Hungary; ^4^Research Center for Natural Sciences, Institute of Cognitive Neuroscience and Psychology, Hungarian Academy of Sciences Budapest, Hungary

**Keywords:** *:* moral superiority, emotional attachment to the in-group, exonerating cognitions, group-based emotions, reparation

## Abstract

Three studies examined how members of a national group react to in-group wrongdoings. We expected that perceived in-group moral superiority would lead to unwillingness to repair the aggression. We also expected that internal-focused emotions such as group-based guilt and group-based shame would predict specific, misdeed-related reparative intentions but not general approach motivation toward the victim groups. In Study 1, facing the in-group’s recent aggression, participants who believed that the Hungarians have been more moral throughout their history than members of other nations, used more exonerating cognitions, experienced less in-group critical emotions and showed less willingness to provide reparations for the members of the victim group. Study 2 and Study 3 confirmed most findings of Study 1. Perceived in-group moral superiority directly or indirectly reduced willingness to provide either general or specific reparations, while internally focused in-group critical emotions predicted specific misdeed-related reparative intentions but not general approach motivation. The role of emotional attachment to the in-group is considered.

## Introduction

“It is striking how quickly the assumption that the angel is analogous with Hungary has gained ground,” Hungarian Prime Minister Viktor Orbán wrote. “I, for instance, see in the angel the innocent victims and not some kind of innocent state.” The Hungarian Prime Minister was talking about the controversial German occupation memorial which was erected during the night of 20 July, 2014. The Nazi German occupiers are represented by a tympanum with a bronze eagle. Below it, there is a bronze statue of the Archangel Gabriel, holding an orb, which is one of the Hungarian national symbols. It is easy to see the intended message of the monument: above there is the perpetrator eagle and below there is the Hungarian angel, the victim. The monument has provoked opposition in Hungary, as many said that it whitewashes the role the Hungarian government and Hungarian people had in the murder of more than half a million Jewish, Romani and gay people during the Holocaust.

There is a growing body of research about the misdeeds committed by the members of the in-group (Branscombe and Doosje, 2004a,b; Wohl et al., 2006; Tropp, 2012). One important question is how innocent group members react to such misdeed? This is particularly interesting when the perpetrator group has a sense of moral superiority (László, 2013) which often provides group members with the license to commit aggression and violence (Sommer and Baumeister, 1998).

Our studies were aimed to demonstrate that group members’ sense of in-group moral superiority originating in the group’s history results in exonerating cognitions, fewer emotional responses to the in-group’s actions and less willingness to compensate the out-group for the harm done. One important objective of the research was to reveal that perceived in-group moral superiority not only reduces reparative intentions related to specific misdeeds but also the general motivation to restore the relations with the concerned out-group. In Studies 2 and 3, we also examined externally focused emotions felt toward the out-group. We hypothesized that such emotions would be more closely related to understanding the out-group’s situation and so to the general approach motivation. Furthermore, we hypothesized that participants regarding the in-group morally superior to the out-groups would be less willing to take the perspective of these groups.

The relationship between perceived moral superiority and reparative intentions was examined by presenting participants with four accounts of intergroup events involving in-group aggression. These accounts were used to assess the use of exonerating cognitions, the experience of group-based emotions and the importance of emotional attachment to the in-group.

We start our paper by briefly reviewing previous research on the in-group’s misdeeds, exonerating cognitions, group-based emotions and reparative behavior. Then, we discuss the concept of self-perceived collective victimhood and its relevance in the Hungarian context. The role of emotional attachment to the in-group is considered. Finally, we present three empirical studies about moral deviations committed by the Hungarian group.

### The In-group’s Misdeeds: Exonerating Cognitions, Group-Based Emotions and Reparative Intentions

People engage in a number of distinct strategies when members of one’s own group commit moral deviations. Group members can decrease their identification with the group (Kessler and Hollbach, 2005) or, alternatively, they can use “black sheep” strategies, the derogation of in-group members who committed the moral deviations (Marques et al., 1988). They can explain away the misdeeds by the implementation of exonerating cognitions (Roccas et al., 2006). Last but not least they can accept the in-group’s responsibility and the accompanying negative emotions (Xu et al., 2011).

A considerable amount of research has been dedicated to identify the common cognitions which can help group members refuse the group’s responsibility and aversive emotions such as collective guilt. Roccas et al. (2006) call these processes exonerating cognitions. Examples of these cognitions are moral justifications, advantageous comparisons, responsibility denial, and out-group blaming. Moral justification is a process whereby detrimental conduct is explained by valued social or moral purposes. Advantageous comparison is a comparison of the moral deviation to an even more serious misdeed, thus making the harmful conduct look good. The denial of responsibility is also a convenient strategy used to reduce negative consequences of moral deviance. Blaming the out-group makes the perpetrator the faultless victims, and portrays the transgression as self-defense (Bandura, 1999; Miller, 2001; Feather, 2006; Xu et al., 2011).

The literature on group-based emotions also suggests that it is possible that group members face the immorality of the in-group (Doosje et al., 1998). People can experience moral emotions such as group-based guilt and group-based shame solely because of their association (i.e., common group membership) with the aggressors. Group-based guilt and shame refer to instances in which people are feeling guilt or shame because of the wrongdoings of fellow-group members (Lickel et al., 2011). One of the main reasons these emotions are so important is their supposed connection with reparative intentions and behaviors such as apology, asking for forgiveness, material compensation and striving for a harmonious relationship with the members of the victim group (Doosje et al., 1998; Swim and Miller, 1999; Iyer et al., 2003; Branscombe et al., 2007; Brown et al., 2008; Giner-Sorolla et al., 2008; Berndsen and McGarty, 2010). However, recent research suggests that group-based guilt and shame does little to meaningfully affect positive attitudes toward reconciliation (McGarty et al., 2005; Halloran, 2007; Pagano and Huo, 2007; Maoz and Ellis, 2008; Lickel et al., 2011; Imhoff et al., 2012). Out-group focused emotions such as empathy, sympathy, and regret (Leach et al., 2006; Imhoff et al., 2012) are often found to have more significant effects on ‘meaningful’ reparation than group-based guilt and shame.

The present studies were aimed at a closer examination of the similarities and differences between various group-based emotions. Following Figueiredo et al. (2015) conception of group-based compunction (an intertwined experience of guilt and self-criticism or shame), we distinguished between in-group focused negative emotions (shame, guilt, and in-group directed anger) on one hand, and out-group focused emotions (empathy, sympathy, and regret) on the other hand. While self-focused emotions may have a limited effect on reparation, emotions focusing on the suffering of others rather than on the misdeed of the in-group is proved to increase prosocial activism, i.e., the real motivation to help others (see, e.g., Ortony et al., 1988; Hoffman, 1991; Batson, 1998; Iyer et al., 2003).

Another equally important question concerns what researchers mean by reparation exactly. Many studies treat different forms of reparation such as offering financial compensation or presenting formal public apologies as interchangeable (e.g., Doosje et al., 1998). However, Zebel et al. (2009) argue that there is a difference between symbolic and material reparation. The same distinction was made by Licata and Klein (2010). Berndsen and McGarty (2010) argues that apology and restitution are two closely connected but distinct concepts and both are different from the importance of a harmonious relationship between groups. In our view, one particularly important and yet scarcely studied difference between different forms of reparation concerns its purpose; more specifically, the reparative behavior may be aimed solely at easing the pain of negative, aversive emotions, or, alternatively, it may be aimed at a more meaningful, more honest approach to the victim group. In our studies, we were interested in the differences between specific misdeed-related reparations and general approach motivation. This distinction between “specific reparation” and “general approach motivation” leads to an important question: are the aversive collective emotions such as group-based guilt and group-based shame as strongly associated with the approach motivation as with the specific reparation gestures?

### Self-perceived Collective Victimhood and Perceived In-group Moral Superiority: The Hungarian Context

National history serves as an interpretive framework for both past and contemporary events and experiences (László, 2013; Pilecki and Hammack, 2014). This is a scarcely studied aspect of the perpetrator-victim intergroup dynamics. According to Bar-Tal et al. (2007), the group’s emotional climate and collective emotional orientation can serve as a “lens through which group members interpret conflictive or peaceful events” (p. 447). The group’s emotional climate and collective emotional orientation largely depends on past collective experiences, that is, on history (Bar-Tal et al., 2007; Halperin and Bar-Tal, 2011).

In our studies, we focused our attention to the Hungarian national group, which according to László (2013) has a historical narrative of constant, undeserved, unjust, and immoral victimization by other groups such as the Ottomans, the Austrians, the German, Nazis, the Soviets, and the neighboring national groups. As a consequence, Hungarians have a vulnerable identity where self-criticism is rare and exceptional and there is a general sense of a lack of agency. According to László (2013), the Hungarian identity is very similar to what Bar-Tal et al. (2009) call a self-perceived victimhood mindset (see also Vollhardt, 2012). The Hungarian context has been studied extensively by László and his colleagues (for a summary, see László, 2013). The analysis of historical textbooks (Fülöp et al., 2014), historical novels (Vincze et al., 2007; László and Somogyváry, 2008), and folk historical narratives (László and Somogyváry, 2008) as well as questionnaire studies (Mészáros et al., 2014) show the same pattern of uncontrollable fall and inevitable victimization of the once glorious Hungarians.

However, the historical truth is that even during their recent history, Hungarians were not only passive sufferers but also active perpetrators in some instances. In our studies, we were interested in the reactions of in-group members to violence which was committed by the members of their in-group and which contradicts the generally accepted victim story of the Hungarians.

We argue that the above perception of the in-group’s history results in the perceived moral superiority of the in-group. Identifying the in-group with the victim and the out-groups with the aggressors may lead in-group members to view their own group as having been morally superior to other groups during the course of history. Participants who feel that the in-group has been more moral throughout its history than other groups would feel challenged when facing the in-group’s aggression: in that situation, they would use exonerating cognitions and avoid both in-group critical emotions and reparation. Furthermore, participants focusing on the in-group’s moral superiority will report lower degree of out-group focused emotions such as empathy, sympathy, and regret toward members of out-groups victimized by the in-group (Chaitin and Steinberg, 2008). We also considered the role of emotional attachment to the in-group. According to Roccas et al. (2006) people who are highly identified in this sense define themselves in terms of their group membership and extend their self-concept to include the group. They feel emotionally attached to the group and want to contribute to it.

## Overview of Current Research

In our studies, we were interested in the relationship between perceived in-group moral superiority and specific misdeed-related reparation and general approach motivation toward the members of the victim group. We used four stories in which members of the Hungarian in-group wronged members of other national groups (see the full stories in Appendices A–D).

The main hypothesis predicted that participants who perceive the in-group as morally superior compared to other nations would report lower degrees of reparative intentions. This would especially apply to general approach motivation which not only concern the wrongdoing in question but describe a more general motivation to recover the relationship with the out-group. We also hypothesized that perceived in-group moral superiority would have its effect on reparative intentions through the use of exonerating strategies and group-based emotions.

The first study aimed to establish the relationship between moral superiority and specific, misdeed-related reparative intentions. The main hypothesis of the study predicted that participants with high scores on moral superiority would be more likely to use exonerating strategies, and they would less likely to experience group-based guilt, shame and in-group directed anger. As a consequence they would be also less willing to compensate and apologize to the out-group.

In the second study, we introduced regret as an out-group focused emotion. Several studies found meaningful differences between internally and externally focused emotions as well as differences in consequences of the two types of emotional experience (Imhoff et al., 2012). Imhoff et al. (2012) suggest that guilt is an internally focused aversive emotion whereas regret is an empathic emotion emerging when one takes the victim’s perspective. Guilt is primarily associated with specific reparation while regret is generally related to the willingness to engage in intergroup contact. Accordingly, we expected that internally focused emotions would primarily accompany specific, event-related reparations while regret would be associated with general approach motivation. Furthermore, we expected moral superiority to be associated with lower degrees of in-group critical emotions as well as less regret in relation to the in-group’s aggression.

The second study used a recent event, the 2009 ice hockey world championship in Switzerland. In the third study, we wanted to replicate the results of the second study using two events which happened in the distant past. In the third study, empathy and sympathy were added to the studied externally focused emotions. We distinguished empathy and sympathy from regret which latter Imhoff et al. (2012) define as a non-aversive externally focused emotion lacking an object (empathy and sympathy toward out-group members vs. regret over the events).

Ethical approval was not required for these studies as per the institutional and national requirements. However, we obtained a retroactive ethics approval.

### Study 1: Atrocities against the Serbian Minorities in Hungary

#### Method

##### Participants

One hundred and sixty-six participants participated in the study (92 female, 74 male; *M*_age_ = 25.58 years, *SD* = 10.3). Most participants were university students at a Hungarian university. Participants were informed that participation in the study was voluntary and anonymous. Participants received the story and the related questionnaires on-line. They received no reward for participation. Participants were thanked and debriefed at the end of the study.

##### Procedure

Participants first completed a measure of perceived moral superiority of the in-group and emotional attachment to the in-group, and then read a one-page account of recent concerning events in which the Hungarians were the perpetrators and the Serbs were the victims. After the account, participants rated statements about group-based emotions, exonerating cognitions, and reparative intentions related to the recounted events. Similarly to the study of Roccas et al. (2006), the description of the events clearly indicated that the Hungarians’ actions were intentional. The recent concerning events involved violence and atrocities against the Serbian minorities in Hungary (e.g., anti-Serbian graffiti, vandalism against a Serbian church, insulting Serbian people, see Appendix A for the whole account).

##### Measures

###### Perceived moral superiority of the in-group and emotional attachment to the in-group

Perceived moral superiority of the in-group was measured with one item (“Compared to other nations, Hungarians have acted more morally throughout their history”). Emotional attachment to the in-group was measured with four items adapted from Roccas et al. (2006). Example item is “Being a Hungarian is an important part of my identity.” Participants indicated their degree of agreement with each item on a 7-point scale ranging from *strongly disagree* (1) to *strongly agree* (7). The emotional attachment to the in-group scale showed good internal consistency (α = 0.85).

###### Exonerating cognitions, group-based emotions, reparative intentions

The one-page summary of recent violence against Serbian minorities in Hungary was followed by 12 items, each rated on a 7-point scale ranging from *completely disagree* (1) to *completely agree* (7).

####### Exonerating cognitions

We used four self-designed items to assess exonerating cognitions: The above account is an accurate description of the actual events (reversed); Even if the account presents the events as they actually happened, they were only reactions to the Serbs’ preceding actions; It primarily is the Serbs’ responsibility that the relations between the two groups developed as they did; The Hungarians’ actions were not determined by external circumstances (reversed). The obtained data were analyzed in such a way that the effect of each exonerating strategy was taken into account separately since the different exonerating strategies were not closely associated with each other (coefficients of correlation between the strategies varied between 0.02 and 0.48). Low and medium correlations between the exonerating cognitions is in part due to the logical relations between the statements being interdependent in terms of applicability: for example, if one finds that the account does not cover historical truth, then one may find that a statement blaming out-group members for the recounted events is inapplicable and this in turn will be indicated by a low degree of agreement on the scale.

####### In-group focused, in-group critical emotions

We used four self-designed items to assess in-group focused, in-group critical emotions related to the event (I feel guilty about the events; When I read about events like these, I feel guilty as a Hungarian; When I read about events like these, I feel ashamed as a Hungarian; I am angry with the Hungarians because of the events; α = 0.84)

####### Reparative intentions

Reparative intentions were measured by four self-designed items (We Hungarians should compensate the Serbs; The Hungarian government owes apologies to the Serbs for the events; The Hungarian group owes apologies to the Serbs for the events; The Hungarian government should compensate the Serbs for the events; α = 0.86)

#### Results

##### Correlations and means

The correlations between all variables under analysis are presented in **Table 1**.

**Table 1:** Descriptive statistics and correlations between measures (*N* = 166).

Measures	1	2	3	4	5	6	7	8
(1) Moral superiority	-	0.43**	-0.27**	0.19*	0.39**	0.00	-0.25**	-0.39**
(2) Emotional attachment		-	-0.19*	0.09	0.18*	-0.13	-0.11	-0.19*
(3) EC1			-	-0.10	-0.26**	0.07	0.48**	0.57**
(4) EC2				-	0.48**	-0.10	-0.18*	-0.21**
(5) EC3					-	-0.02	-0.45**	-0.42**
(6) EC4						-	0.13	0.15
(7) In-group critical emotions							-	0.76**
(8) Reparative intentions								-
*M*	4.13	4.94	4.27	4.00	3.60	3.66	4.46	3.99
*SD*	1.57	1.33	1.64	1.70	1.50	1.45	1.49	1.48


*EC1, The above account is an accurate description of the actual events (Reversed); EC2, even if the account presents the events as they actually happened, they were only reactions to the Serbs’ preceding actions; EC3, it primarily is the Serbs’ responsibility that the relations between the two groups developed as they did; EC4, the Hungarians’ actions were not determined by external circumstances (Reversed). ^∗^*p* < 0.05, ^∗∗^*p* < 0.01, two-tailed tests.*

**FIGURE 1:**
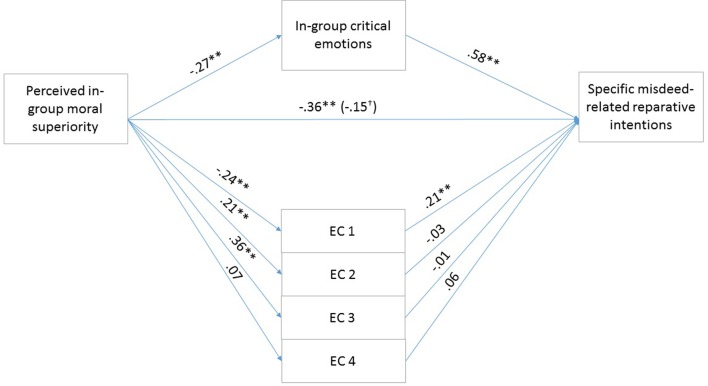
Results for the multiple mediation model in Study 1. For each of the mediators, the unstandardized path coefficients are indicated. The line connecting perceived in-group moral superiority with specific misdeed-related reparative intentions indicates the total effect of moral superiority on specific misdeed-related reparative intentions (direct effect of moral superiority on specific misdeed-related reparative intentions is in the parentheses). EC1, the above account is an accurate description of the actual events (Reversed); EC2, even if the account presents the events as they actually happened, they were only reactions to the Serbs’ preceding actions; EC3, it primarily is the Serbs’ responsibility that the relations between the two groups developed as they did; EC4, the Hungarians’ actions were not determined by external circumstances (Reversed). *^∗^ p* < 0.05; *^∗∗^ p* < 0.01.

To test our hypotheses, that the exonerating cognitions and the in-group critical, in-group focused emotions mediate the effects of perceived in-group moral superiority on reparative intentions, we conducted a parallel multiple mediation analysis using Model 4 of the PROCESS macro offered by Hayes (2013). The mediation analysis examined the indirect effects of perceived in-group moral superiority on specific, misdeed-related reparative intentions through exonerating cognitions and in-group critical, in-group focused emotions, controlling for emotional attachment to the in-group. We calculated indirect effects using 5000 bootstrap iterations. If the bias-corrected 95% CI does not contain zero, the indirect effect is considered to be significant. Unstandardized regression coefficients of the direct effects are reported in **Figure 1**.

Results were in line with our predictions. The overall model was significant (*F*_7,158_ = 44.28, *p* < 0.01) and explained 66.2% of the variance in reparation intention scores. The total effect of perceived in-group moral superiority on reparation intention was significant (*b* = -0.3568, *SE* = 0.0753, *t* = -4.7357, *p* < 0.01, 95% CIs = -0.5056, -0.2080). The covariate emotional attachment to the in-group was not significant (*p* = 0.85). The 95% confidence intervals for the mediator variables are shown in **Table 2**. The indirect effect of perceived in-group moral superiority on reparative intentions through Exonerating cognition 1 and in-group critical, in-group focused emotions were significant. Finally, the direct effect of perceived in-group moral superiority on reparative intentions remained significant [*b* = -0.1459, *SE* = 0.0523, *t* = -2.7920, *p* < 0.01, 95% CI (-0.2491, -0.0427)], therefore indicating partial mediation by exonerations and emotions.

**Table 2:** Ninety-five percent confidence intervals for the mediator variables in Study 1.

		Bootstrapped 95% CIs	
		
Dependent variable	Mediators	Lower	Upper
Reparation intention	EC1	-0.1104	-0.0127
	EC2	-0.0389	0.0093
	EC3	-0.0477	0.0390
	EC4	-0.0039	0.0298
	In-group critical emotions	-0.2661	-0.0546
	Total	-0.3394	-0.0795


*EC1, The above account is an accurate description of the actual events (Reversed); EC2, even if the account presents the events as they actually happened, they were only reactions to the Serbs’ preceding actions; EC3, it primarily is the Serbs’ responsibility that the relations between the two groups developed as they did; EC4, the Hungarians’ actions were not determined by external circumstances (Reversed).*

#### Discussion

The results show that participants believing in the moral superiority of the in-group were less willing to compensate the out-group for the in-group’s aggression. Perceived moral superiority has both direct and indirect effects on reparative intention. Participants holding the view of a morally superior in-group use more exonerating strategies and experience less in-group critical emotions when facing the in-group’s aggression. Reparative intention is prevented by exonerating strategies while facilitated by in-group critical emotions. Emotional attachment to the in-group shows no relationship with reparative intentions. We included two additional variables in the second study according to the considerations discussed in the “Introduction”: one was regret as an externally focused emotion and the other was the general motivation to recover the relationship with the out-group (striving for a good relationship). We hypothesized that participants who have high scores on perceived in-group moral superiority would report lower degrees of regret while regret would be a good predictor of both general and specific reparative intentions (Imhoff et al., 2012). Furthermore, we hypothesized that while in-group focused, in-group critical emotions would be good predictor of specific reparative intentions directly related to the in-group’s aggression (apology, material compensation), they would show a weaker relationship with general approach motivation. This latter hypothesis is in line with empirical results reported in recent years which point out that guilt and shame have limited importance in a meaningful recovery of intergroup relations (McGarty et al., 2005; Halloran, 2007; Pagano and Huo, 2007; Maoz and Ellis, 2008; Lickel et al., 2011; Imhoff et al., 2012).

### Study 2: Outrageous Hungarian Supporters at the 2009 Ice Hockey World Championships

#### Method

##### Participants

One hundred and one participant participated in the study (64 female, 37 male; *M*_age_ = 20.20 years, *SD* = 2.57). Participants were students at a Hungarian university. Participants were informed that participation in the study was voluntary and anonymous. Participants received no reward for participation. Participants were thanked and debriefed at the end of the study in groups.

##### Procedure

Participants first completed a measure of perceived in-group moral superiority and emotional attachment to the in-group, and then read an account of about half a page in length presenting a group-stage game at the 2009 Ice Hockey World Championships between the Hungarian and Slovak national ice hockey teams. According to the account, Hungarian supporters first jeered the Slovak national anthem and then chanted anti-Slovak slogans. Several Hungarian supporters had to be forced out of the stadium. After the match, Hungarian supporters beat a Slovak supporter (See Appendix B for the full account of the event). After reading the account, participants rated statements about group-based emotions, exonerating cognitions, and reparative intentions related to the recounted events. The description of the event clearly indicated that the Hungarians’ actions were intentional.

##### Measures

###### Perceived moral superiority of the in-group and emotional attachment to the in-group

Perceived moral superiority of the in-group and emotional attachment to the in-group were measured with the same items as in Study 1. The emotional attachment to the in-group scale showed acceptable internal consistency (α = 0.73).

###### Exonerating cognitions, group-based emotions, reparative intentions

The half-page summary of the ice hockey game was followed by 13 items, each rated on a 7-point scale ranging from *completely disagree* (1) to *completely agree* (7). Exonerating cognitions were measured by six items. In addition to the four items, which we used in Study 1, we added 2 new items: “The account of the event is too harsh”; “The Hungarians treated the Slovaks unjustly.” Three items tapped in-group focused, in-group critical emotions (“I feel guilty about the events”; When I read about events like these, I feel ashamed as a Hungarian”; “I am angry with the Hungarians because of the events”; α = 0.77). Regret was measured with one item (“I feel regret over the events”). In this study, we distinguished specific forms of reparative intentions from a more general motivation to maintain a good relationship with out-group members. Specific, event-related reparative intentions were measured by two items (We Hungarians should compensate the Slovaks; We Hungarians should offer an apology to the Slovaks, *r* = 0.45), and general approach motivation was measured by one item adapted from Berndsen and McGarty (2010) (“I think that we, the Hungarians should strive for a harmonious relationship with the Slovaks”).

#### Results

##### Correlations and means

The correlations between all variables under analysis are presented in **Table 3**.

**Table 3:** Descriptive statistics and correlations between measures (*N* = 101).

Measures	1	2	3	4	5	6	7	8	9	10	11	12
(1) MS	-	0.27**	0.02	0.13	0.40**	0.11	-0.23*	-0.06	-0.08	-0.06	-0.26**	-0.25*
(2) EA		-	-0.05	0.27**	0.30**	-0.03	-0.06	0.07	-0.01	0.07	-0.10	0.10
(3) EC1			-	0.14	-0.02	0.17	-0.03	-0.07	-0.03	0.05	-0.11	-0.14
(4) EC2				-	0.27**	0.45**	-0.46**	-0.24*	-0.27**	-0.15	-0.36**	-0.34**
(5) EC3					-	0.17	-0.36**	-0.07	-0.03	0.08	-0.36**	-0.12
(6) EC4						-	-0.52**	-0.24*	-0.28**	-0.09	-0.39**	-0.50**
(7) EC5							-	0.16	0.48**	0.22*	0.71**	0.56**
(8) EC6								-	0.12	0.19	0.08	0.02
(9) IE									-	0.49**	0.48**	0.33**
(10) Regret										-	0.23*	0.15
(11) Specific											-	0.52**
(12) General												-
*M*	3.60	5.04	3.61	3.49	3.96	3.13	5.02	4.17	4.42	4.24	4.26	4.54
*SD*	1.44	1.86	1.70	1.74	2.08	1.96	1.84	1.69	1.74	1.91	1.61	1.95


*MS, moral superiority; EA, emotional attachment; IE, in-group critical emotions; Specific, specific, misdeed-related reparations; General, general approach motivation; EC1, the above account is an accurate description of the actual events (Reversed); EC2, the account of the event is too harsh; EC3, even if the account presents the events as they actually happened, they were only reactions to the Slovaks’ preceding actions; EC4, it primarily is the Slovaks’ responsibility that the relations between the two groups developed as they did; EC5, the Hungarians treated the Slovaks unjustly (Reversed); EC6, the Hungarians’ actions were not determined by external circumstances (Reversed). ^∗^*p* < 0.05, ^∗∗^*p* < 0.01, two-tailed tests.*

**FIGURE 2:**
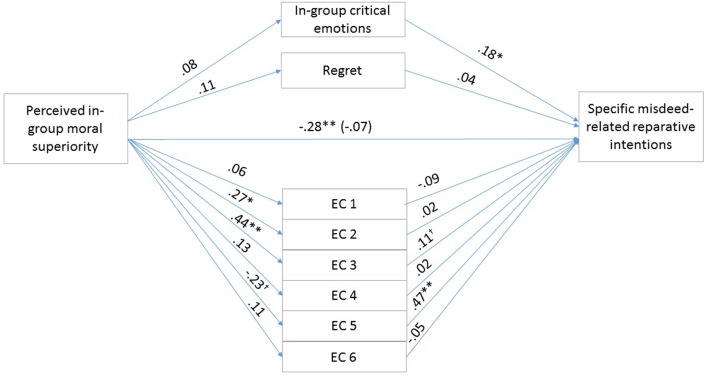
Results for the multiple mediation model in Study 2. For each of the mediators, the unstandardized path coefficients are indicated. The line connecting perceived in-group moral superiority with specific misdeed-related reparative intentions indicates the total effect of moral superiority on specific misdeed-related reparative intentions (direct effect of moral superiority on specific misdeed-related reparative intentions is in the parentheses). EC1, the above account is an accurate description of the actual events (Reversed); EC2, the account of the event is too harsh; EC3, even if the account presents the events as they actually happened, they were only reactions to the Slovaks’ preceding actions; EC4, it primarily is the Slovaks’ responsibility that the relations between the two groups developed as they did; EC5, the Hungarians treated the Slovaks unjustly (Reversed); EC6, the Hungarians’ actions were not determined by external circumstances (Reversed). *^∗^p* < 0.05; *^∗∗^p* < 0.01.

**FIGURE 3:**
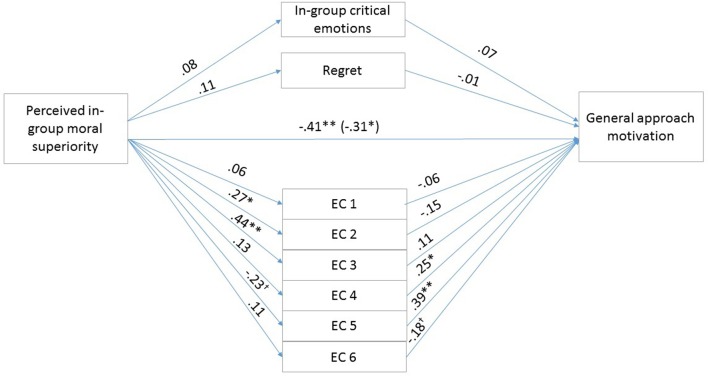
Results for the multiple mediation model in Study 2. For each of the mediators, the unstandardized path coefficients are indicated. The line connecting perceived in-group moral superiority with general approach motivation indicates the total effect of moral superiority on general approach motivation (direct effect of moral superiority on general approach motivation is in the parentheses). EC1, the above account is an accurate description of the actual events (Reversed); EC2, the account of the event is too harsh; EC3, even if the account presents the events as they actually happened, they were only reactions to the Slovaks’ preceding actions; EC4, it primarily is the Slovaks’ responsibility that the relations between the two groups developed as they did; EC5, The Hungarians treated the Slovaks unjustly (Reversed); EC6, The Hungarians’ actions were not determined by external circumstances (Reversed). *^∗^p* < 0.05; *^∗∗^p* < 0.01.

To test our hypotheses, that the exonerating cognitions, the in-group critical, in-group focused emotions, and regret mediate the effects of perceived in-group moral superiority on general and specific reparative intentions, we conducted parallel multiple mediation analyses using Model 4 of the PROCESS macro offered by Hayes (2013). We calculated indirect effects using 5000 bootstrap iterations. Unstandardized regression coefficients of the direct effects are reported in **Figures 2**, **3**.

The first mediation analysis examined the indirect effects of perceived in-group moral superiority on specific, misdeed-related reparative intentions through exonerating cognitions, and group-based emotions, controlling for emotional attachment to the in-group. The overall model was significant (*F*_10,90_ = 11.63, *p* < 0.01) and explained 56.37% of the variance in specific, misdeed-related reparative intentions. The total effect of perceived in-group moral superiority on reparation intention was significant (*b* = -0.2872, *SE* = 0.1134, *t* = -2.5334, *p* < 0.05, 95% CIs = -0.5122, -0.0622). The covariate emotional attachment to the in-group was not significant (*p* = 0.79). The 95% confidence intervals for the mediator variables are shown in **Table 4**. The indirect effect of perceived in-group moral superiority on reparative intentions through Exonerating cognition 5 was significant. Finally, the direct effect of perceived in-group moral superiority on reparative intentions was not significant [*b* = -0.0697, *SE* = 0.0876, *t* = -0.7959, *p* > 0.05, 95% CI (-0.2437, 0.1043)], therefore indicating full mediation by Exonerating cognition 5. The second mediation analysis examined the indirect effects of perceived in-group moral superiority on general approach motivation through exonerating cognitions, and group-based emotions, controlling for emotional attachment to the in-group. Unstandardized regression coefficients of the direct effects are reported in **Figure 3**. The overall model was significant (*F*_10,90_ = 7.6999, *p* < 0.01) and explained 46.11% of the variance in general approach motivation. The total effect of perceived in-group moral superiority on general approach motivation was significant (*b* = -0.4119, *SE* = 0.1351, *t* = -3.0492, *p* < 0.05, 95% CIs = -0.6800, -1438). The covariate emotional attachment to the in-group was marginally significant (*b* = 0.1971, *SE* = 0.1047, *t* = 1.8825, *p* = 0.0627, 95% CIs = -0.0107, 0.4050). The 95% confidence intervals for the mediator variables are shown in **Table 4**. The indirect effect of perceived in-group moral superiority on general approach motivation through Exonerating cognition 5 was significant. Finally, the direct effect of perceived in-group moral superiority on general approach motivation remained significant [*b* = -0.3071, *SE* = 0.1177, *t* = -2.6098, *p* < 0.05, 95% CI (-0.5408, -0.0733)], therefore indicating partial mediation by exonerations.

**Table 4:** Ninety-five percent confidence intervals for the mediator variables in Study 2.

		Bootstrapped 95% CIs	
		
Dependent variable	Mediators	Lower	Upper
Specific reparation intention	EC1	-0.0562	0.0165
	EC2	-0.0174	0.0485
	EC3	-0.1494	0.0215
	EC4	-0.0716	0.0250
	EC5	-0.3123	-0.0185
	EC6	-0.0096	0.0614
	In-group critical emotions	-0.1020	0.0173
	Regret	-0.0561	0.0146
	Total	-0.4247	-0.0310
General approach motivation	EC1	-0.0640	0.0217
	EC2	-0.1095	0.0185
	EC3	-0.0317	0.1812
	EC4	-0.1896	0.0238
	EC5	-0.3172	-0.0097
	EC6	-0.0148	0.1122
	In-group critical emotions	-0.0826	0.0165
	Regret	-0.0279	0.0631
	Total	-0.3523	0.1304


*EC1, The above account is an accurate description of the actual events (Reversed); EC2, the account of the event is too harsh; EC3, even if the account presents the events as they actually happened, they were only reactions to the Slovaks’ preceding actions; EC4, it primarily is the Slovaks’ responsibility that the relations between the two groups developed as they did; EC5, The Hungarians treated the Slovaks unjustly (Reversed); EC6 = the Hungarians’ actions were not determined by external circumstances (Reversed). ^∗^*p* < 0.05, ^∗∗^*p* < 0.01, two-tailed tests.*

#### Discussion

Study 2 has revealed that reparative intentions specifically related to the misdeed are primarily determined by the perceived moral superiority of the in-group. Perceived moral superiority of the in-group prevents reparative intentions both directly and indirectly, through exoneration of the in-group. This finding is similar to the results obtained in Study 1. Perceived in-group moral superiority, exoneration of the in-group and in-group focused, in-group critical emotions showed the same relationship with specific, misdeed-related reparative intentions. However, perceived in-group moral superiority was not associated with the experience of in-group focused, in-group critical emotions in this study. Regarding regret, our hypothesis was not confirmed by the results: regret did not predict either specific reparative intentions or general approach motivation, nor showed a relationship with perceived in-group morality. To our view, this finding does not contradict those reported by Imhoff et al. (2012) since they measured regret in response to events of a larger scale whereas our study only focused on a single episode of an intergroup conflict. In any case, comparing the results of our studies with those reported by Imhoff et al. (2012) point to the need of further investigations into regret. Emotional attachment to the in-group, however, had an interesting role in our study: while it showed no relationship with misdeed-related specific reparation in harmony with findings of Study 1, it had a marginally significant direct effect on striving for a good relationship. Apparently, participants with high emotional attachment to the in-group and low levels of perceived in-group moral superiority assign more importance to the recovery of intergroup relations. With regard to current theoretical debates in the literature, probably the most remarkable result of Study 2 is that while the scale measuring group-based guilt, group-based shame and in-group directed anger was a good predictor of specific reparative intentions, it showed no significant relationship with general approach motivation. These results corroborate the conclusion drawn from previous studies suggesting that such emotions have limited implications for a meaningful recovery of intergroup relations (McGarty et al., 2005; Brown and Cehajic, 2008; Imhoff et al., 2012).

Study 1 and Study 2 used events which took place in the recent past. Participants could easily find exonerations for these events on the basis that the cases of Hungarian aggression (graffiti, outrageous supporters) were insignificant compared to the actions of the out-groups ending and following WWI, and the former were only reactions to all the wrong the Hungarians had to endure after the dissolution of pre-Trianon Hungary. Study 3 used two events which took place before WWI: an atrocity committed against Slovak villagers and repression of a Croatian university student protest. Both events took place in pre-Trianon Hungary and Hungarians as the dominant group committed aggression against out-groups which formed minorities in contemporary Hungary. We aimed to establish whether these events would elicit the same mechanisms as those revealed in Study 1 and Study 2. In this study, externally focused emotions also included empathy and sympathy toward the victimized out-group besides regret.

### Study 3: Aggression by the Hungarian Gendarmerie Toward Slovak Villagers and Croatian University Students

#### Method

##### Participants

Seven hundred and fifteen participants participated in the study (453 female, 262 male; *M*_age_ = 26.45 years, *SD* = 10.27). Participation was voluntary and anonymous participants received no reward for participation. Participants were thanked and debriefed at the end of the study.

##### Procedure

Participants first completed a measure of perceived in-group superiority and emotional attachment to the in-group. After that, they were presented with two accounts. After each account, participants rated statements about exonerating cognitions, group-based emotions and reparative intentions. The descriptions of the events clearly indicated that the Hungarians’ actions were intentional and their consequences were foreseeable.

One of the accounts presented the Černová clash. In 1907, during a church consecration in the Slovak village Černová, the gendarmes came into conflict with the villagers. The gendarmes drove a carriage into the crowd that in response attacked the gendarmes who then fired at the crowd. Nine people died immediately, six died in the following days and many were wounded severely or slightly. The other recounted event took place in 1890. The Hungarian National Assembly passed a state language law which prescribed the mandatory use of Hungarian language in the entire territory of the country. Croatian university students protested in the streets that the Hungarian gendarmerie attempted to disperse. Students’ resistance to leaving led to a violent scrimmage and finally the gendarmes broke up the crowd severely wounding several protesters (see Appendices C, D for the whole account of the events).

The order of presentation of the accounts was counterbalanced: one half of participants were first presented with the account of the Černová clash while the others first read about the Croatian protest.

##### Measures

###### Perceived in-group superiority and emotional attachment to the in-group

The same items were used as in Study 1 and Study 2 to measure perceived in-group moral superiority and emotional attachment to the in-group (α = 0.80 for the emotional attachment scale).

###### Exonerating cognitions, group-based emotions, reparative intentions

Each of the two stories was followed by the same 15 items, each rated on a 7-point scale ranging from *completely disagree* (1) to *completely agree* (7). Exonerating cognitions, in-group focused, in-group critical emotions, regret, specific misdeed-related reparations and general approach motivation were measured by the same items as in Study 2. Sympathy and empathy toward the out-groups were also measured (“I feel sympathy for the Croatian victims of the story”; “I feel empathy for the Croatian victims of the story”).

#### Results

##### Correlations, means and reliability statistics.

The correlations between all variables under analysis are presented in **Table 5**.

**Table 5:** Descriptive statistics and correlations between measures (*N* = 715).

Measures	1	2	3	4	5	6	7	8	9	10	11	12	13
(1) MS	-	0.42**	-0.07	0.30**	-0.07	0.32**	-0.15**	0.23**	0.03	0.02	-0.07	-0.03	-0.13^∗∗^
(2) EA	0.42**	-	-0.01	0.16**	-0.03	0.13**	-0.04	0.15**	0.02	0.10**	0.02	-0.03	0.05
(3) EC1	-0.04	0.03	-	-0.22**	0.07	-0.07	0.12**	-0.14**	0.15**	0.15**	0.20**	0.06	0.09*
(4) EC2	0.34**	0.17**	-0.15**	-	0.04	0.31**	-0.05	0.35**	0.02	-0.03	-0.08*	-0.10**	-0.16**
(5) EC3	-0.06	-0.04	0.03	-0.01	-	-0.04	0.60**	-0.15**	0.35**	0.29**	0.33**	0.36**	0.16**
(6) EC4	0.34**	0.17**	0.01	0.33**	-0.04	-	-0.15**	0.42**	-0.02	-0.04	-0.15**	-0.10*	-0.25**
(7) EC5	-0.07	-0.01	0.03	-0.03	0.66**	-0.07	-	-0.25**	0.45**	0.36**	0.45**	0.50**	0.32**
(8) EC6	0.32**	0.14**	-0.04	0.33**	-0.21**	0.48**	-0.21**	-	-0.08*	-0.11**	-0.25**	-0.07	-0.29**
(9) IE	0.07	0.05	0.06	0.02	0.43**	0.01	0.52**	-0.14**	-	0.58**	0.58**	0.53**	0.27**
(10) Regret	0.04	0.12**	0.06	-0.04	0.32**	-0.03	0.43**	-0.11**	0.59**	-	0.51**	0.34**	0.32**
(11) OE	-0.06	0.05	0.08*	-0.07	0.41**	-0.17**	0.51**	-0.26**	0.57**	0.51**	-	0.41**	0.35**
(12) Specific	-0.04	-0.02	0.04	0.02	0.39**	-0.03	0.47**	-0.12**	0.57**	0.36**	0.44**	-	0.30**
(13) General	-0.13**	0.06	0.05	-0.16^∗∗^	0.26**	-0.30**	0.31**	-0.33**	0.23**	0.26**	0.35**	0.31**	-


*The values above the diagonal are the correlations for the Croatian university students story. The values below the diagonal are the correlations for the Slovakian villagers story. MS, Moral superiority; EA, emotional attachment; IE, in-group critical emotions; Specific, specific, misdeed-related reparations; General, general approach motivation; OE, out-group focused emotions; EC1, the above account is an accurate description of the actual events (Reversed); EC2, the account of the event is too harsh; EC3, the Hungarians treated the Croats/Slovaks unjustly (Reversed); EC4, even if the account presents the events as they actually happened, they were only reactions to the Croats’/Slovaks’ preceding actions; EC5, the Hungarians’ actions were not determined by external circumstances (Reversed); EC6, it primarily is the Croats’/Slovaks’ responsibility that the relations between the two groups developed as they did. *^∗^p* < 0.05, ^∗∗^*p* < 0.01, two-tailed tests.*

**FIGURE 4:**
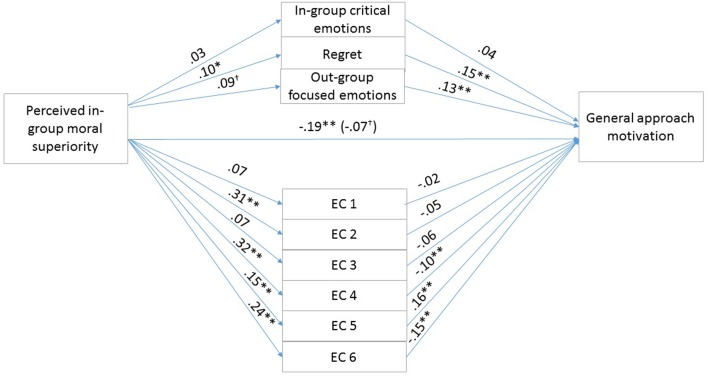
Results for the multiple mediation model in Study 3 Croatian university students story. For each of the mediators, the unstandardized path coefficients are indicated. The line connecting perceived in-group moral superiority with general approach motivation indicates the total effect of moral superiority on general approach motivation (direct effect of moral superiority on general approach motivation is in the parentheses). EC1, the above account is an accurate description of the actual events (Reversed); EC2, the account of the event is too harsh; EC3, the Hungarians treated the Croats unjustly (Reversed); EC4, even if the account presents the events as they actually happened, they were only reactions to the Croats’ preceding actions; EC5, the Hungarians’ actions were not determined by external circumstances (Reversed); EC6, It primarily is the Croats’ responsibility that the relations between the two groups developed as they did. *^∗^p* < 0.05; *^∗∗^p* < 0.01.

**FIGURE 5:**
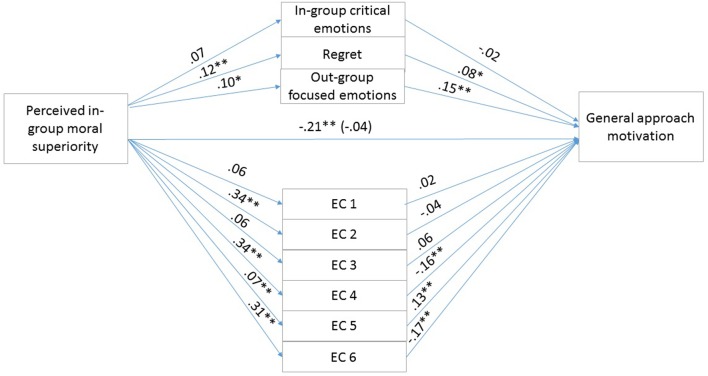
Results for the multiple mediation model in Study 3 Slovakian villagers story. For each of the mediators, the unstandardized path coefficients are indicated. The line connecting perceived in-group moral superiority with general approach motivation indicates the total effect of moral superiority on general approach motivation (direct effect of moral superiority on general approach motivation is in the parentheses). EC1, the above account is an accurate description of the actual events (Reversed); EC2 = the account of the event is too harsh; EC3, the Hungarians treated the Slovaks unjustly (Reversed); EC4, even if the account presents the events as they actually happened, they were only reactions to the Slovaks’ preceding actions; EC5, the Hungarians’ actions were not determined by external circumstances (Reversed); EC6, it primarily is the Slovaks’ responsibility that the relations between the two groups developed as they did. *^∗^p* < 0.05; *^∗∗^p* < 0.01.

To test our hypotheses, that the exonerating cognitions, the in-group critical, in-group focused emotions, regret, and out-group focused emotions mediate the effects of perceived in-group moral superiority on general and specific reparative intentions, we conducted similar parallel multiple mediation analyses as in Study 2. We used Model 4 of the PROCESS macro offered by Hayes (2013). We calculated indirect effects using 5000 bootstrap iterations. **Figures 4**, **5** show the unstandardized regression coefficients of the direct effects for general approach motivation (the total effect of perceived moral superiority on specific, misdeed-related reparative intentions were not significant for either story).

The first mediation analyses examined the indirect effects of perceived in-group moral superiority on specific, misdeed-related reparative intentions through exonerating cognitions, and group-based emotions, controlling for the emotional attachment to the in-group. The overall model was significant for both stories (*F*_11,703_ = 41.10, *p* < 0.01, *R*^2^ was 39.14% for the Croatian university students story and *F*_11,702_ = 39.57, *p* < 0.01, *R*^2^ was 38.27% for the Slovakian villagers story). The total effect of perceived in-group moral superiority on reparation intention was not significant (*p* = 0.67 for the Croatian university student story and *p* = 0.31 for the Slovakian villagers story). The covariate attachment to the in-group was not significant (*p* = 0.65 for the Croatian university student story and *p* = 0.93 for the Slovakian villagers story).

The second mediation analyses examined the indirect effects of perceived in-group moral superiority on general approach motivation through exonerating cognitions, and group-based emotions, controlling for emotional attachment to the in-group. The overall model was significant for both stories (*F*_11,703_ = 20.08, *p* < 0.01, *R*^2^ was 23.91% for the Croatian university students story and *F*_11,702_ = 20.92, *p* < 0.01, *R*^2^ was 24.69% for the Slovakian villagers story). The total effect of perceived in-group moral superiority on general approach motivation was significant (*b* = -0.1929, *SE* = 0.0428, *t* = -4.5029, *p* < 0.05, 95% CIs = -0.2770, -0.1088 for the Croatian university student story and *b* = -0.2104, *SE* = 0.0453, *t* = -4.6409, *p* < 0.05, 95% CIs = -0.2994, -0.1214 for the Slovakian villagers story). The covariate emotional attachment to the in-group was also significant (*b* = 0.1484, *SE* = 0.0477, *t* = 3.1088, *p* < 0.05, 95% CIs = 0.0547, 0.2422 for the Croatian university student story and *b* = 0.1789, *SE* = 0.0505, *t* = 3.5457, *p* < 0.05, 95% CIs = 0.0798, 0.2780 for the Slovakian villagers story). The 95% confidence intervals for all the mediator variables are in **Table 6**. The indirect effect of perceived in-group moral superiority on general approach motivation through Exonerating cognitions 4, 5, 6 and out-group focused emotions was significant for both the Croatian university student and the Slovakian villagers story. Finally, the direct effect of perceived in-group moral superiority on general approach motivation was not significant [*b* = -0.0723, *SE* = 0.0411, *t* = -1.7566, *p* > 0.05, 95% CI (-0.1530, 0.0085) for the Croatian university student story and *b* = -0.0474, *SE* = 0.0440, *t* = -1.0775, *p* > 0.05, 95% CI (-0.1338, 0.0390) for the Slovakian villagers story], therefore indicating full mediation by exonerations and out-group focused emotions.

**Table 6:** Ninety-five percent confidence intervals for the mediator variables in Study 3.

		Bootstrapped 95% CIs	
		
Dependent variable	Mediators	Lower	Upper
General approach motivation Croatian university students story	EC1	-0.0044	0.0129
	EC2	-0.0410	0.0080
	EC3	-0.0013	0.0188
	EC4	-0.0730	-0.0093
	EC5	-0.0556	-0.0098
	EC6	-0.0571	-0.0107
	In-group critical emotions	-0.0023	0.0111
	Regret	-0.0220	0.0097
	Out-group focused emotions	-0.0345	-0.0022
	Total	-0.1741	-0.0716
General approach motivation Slovakian villagers story	EC1	-0.0102	0.0032
	EC2	-0.0406	0.0153
	EC3	-0.0183	0.0015
	EC4	-0.0997	-0.0339
	EC5	-0.0350	-0.0001
	EC6	-0.0930	-0.0243
	In-group critical emotions	-0.0123	0.0044
	Regret	-0.0160	0.0053
	Out-group focused emotions	-0.0376	-0.0029
	Total	-0.2242	-0.1084


*EC1, The above account is an accurate description of the actual events (Reversed); EC2, the account of the event is too harsh; EC3, the Hungarians treated the Croats/Slovaks unjustly (Reversed); EC4, even if the account presents the events as they actually happened, they were only reactions to the Croats’/Slovaks’ preceding actions; EC5, the Hungarians’ actions were not determined by external circumstances (Reversed); EC6, it primarily is the Croats’/Slovaks’ responsibility that the relations between the two groups developed as they did.^∗^*p* < 0.05, *^∗∗^p* < 0.01, two-tailed tests.*

#### Discussion

The results of Study 3 obtained in a historical context of the farther past in part confirmed the results of Study 2. Perceived in-group moral superiority was not related to specific, misdeed-related reparation in either story. Results obtained for general approach motivation were similar to those obtained in Study 2. The effect of perceived in-group moral superiority on general approach motivation was fully mediated by different exonerating cognitions and out-group focused emotions. Emotional attachment to the group was also significantly related to general approach motivation in both stories.

## General Discussion

In this series of studies we examined events in which Hungarians committed aggression against out-groups. Accounts of Hungarian aggression contradict Hungarians’ wide-spread belief that their national in-group has been victimized throughout history. This belief is reflected in a characteristic historical trajectory revealed by László (2013) in which Hungarians are depicted as victims who have been unjustly, immorally, and undeservedly wronged by out-groups throughout history. Studies conducted by László and his research team (for a summary see László, 2013) have demonstrated that relatively little activity is attributed to Hungarians in this trajectory: they are sufferers of, rather than actors in, their own history. This perception of history ignores the fact that sometimes the Hungarians were not the sufferers but the aggressors.

We presented participants with accounts in which clearly the Hungarians were the aggressors and the aggression was intentional. Victims of the aggression were in most cases groups which are traditionally viewed as perpetrators by the Hungarians.

Our primary question was how participants who think that Hungarians have been more moral throughout their history compared to the members of other nation would react to these accounts of Hungarian aggression. The results suggests that perceived in-group moral superiority leads both directly and indirectly, primarily through the use of exonerating cognitions, to unwillingness to repair the aggression by apology or compensation and to unwillingness to strive for a good relationship. Although this result was not obtained for all accounts (see Study 3), yet the pattern seems clear: perceived in-group moral superiority was a significant negative predictor of various forms of recovery of the intergroup relations in 5 of the 6 cases. Perceived in-group moral was primarily related to the use of exonerating strategies, that is, participants who believed in the moral superiority of the in-group interpreted events in a different way than others. Although, our participants used different kind of exonerations for different stories, the pattern seems clear: perceived moral superiority is related to the use of exonerating cognitions, and perceived moral superiority is related to the avoidance of negative group-based emotions and various forms of recovery through exonerations. Our participants questioned the veracity of the historical accounts and viewed Hungarian aggression as a reaction to the out-group’s previous misdeeds, or used biased comparisons. Perceived in-group moral superiority may pose a serious obstacle to the recovery of intergroup relations: exonerating strategies enable group members to maintain the image of a morally superior in-group, however, they do not help to break up with another aspect of such a homogeneous perception of history, namely, that Hungarians have always been the victims of unjust aggression while the perpetrators have always been other groups. Perceived in-group moral superiority not only affects exonerating cognitions: the more recent historical events presented in Study 1 and Study 2 led participants who perceived the in-group as morally superior to refuse in-group critical emotions while events of the far past presented in Study 3 failed to elicit empathy and sympathy in them. Emotional attachment to the in-group has an ambiguous role in the meaningful recovery of intergroup relations: highly attached participants accepted the importance of striving for a good relationship with the out-groups whereas they were not more critical to the in-group’s aggression than less attached participants. Hungarian participants did not show the critical loyalist attitude which was revealed by Roccas et al. (2006) in an Israeli sample: in our studies, attachment showed no relationship with in-group critical emotions, that is, highly attached participants did not report more group-based guilt, group-based shame or in-group directed anger. Only one significant relationship was found between attachment and emotional experience: in Study 3, attachment showed moderate positive correlation with regret. One potential avenue of future research is to focus on identity fusion instead of emotional attachment to the in-group. Identity fusion is a visceral sense of “oneness” with a group and its individual members that motivates personally costly, pro-group behaviors. Fused persons are more inclined to engage in extraordinary behaviors in the service of their group memberships (Swann et al., 2009, 2012, 2014; Swann and Buhrmester, 2015). As identity fusion is more extreme than simple emotional attachment to the group, it would be interesting to see how fused persons would react to in-group misdeeds. Would they engage more or less exonerating cognitions? Would they display more or less group-based emotions? These are very interesting research questions for the future.

Besides the investigation into the relationship between perceived in-group moral superiority and the in-group’s aggression, we also hold important to address current theoretical debates related to the experience of group-based emotions. Probably one of the most important debates concerns collective guilt or, more broadly speaking, in-group critical moral emotions. The literature on collective guilt initially suggested that collective guilt and reparative behavior were clearly related (Doosje et al., 1998; Swim and Miller, 1999; Branscombe et al., 2002). However, studies published in recent years in part questioned this relationship: several studies found that other emotions facilitated reparative behavior more effectively (Lickel et al., 2011) and that guilt as an aversive emotion with low activity had little importance in meaningful reparation (Imhoff et al., 2012). What is more, Imhoff et al. (2012) argue that guilt may even lead the experiencer to distance themselves from the victimized out-group since the aversive feelings elicited by out-group members may deter the experiencer from engaging in intergroup contact. Group-based shame was found to be accompanied by mere one-off reparation gestures aimed at reducing the distress caused by negative emotions, but the possible failure of such an attempt may soon end in withdrawal from the “uncomfortable” relationship (Brown and Cehajic, 2008). We found a consistent pattern: the variable composed of group-based guilt, group-based shame and in-group directed anger was a good predictor of misdeed-related specific reparative intentions in all three studies whereas it did not prove to be a significant positive predictor of general approach motivation in any of the studies. These findings corroborate previous results pointing out the limited importance of guilt or, more generally, in-group focused, in-group critical emotions in the consolidation of positive intergroup relations and in the facilitation of intergroup contact (Maoz and Ellis, 2008).

The role of regret was found to be less clear: it did not predict either specific misdeed-related reparation or general approach motivation in response to more recent events whereas it was positively related to both specific and general reparative intentions in the context of the far past. Regrettably, little empirical knowledge is available concerning regret: our results suggest that regret differs both from internally focused aversive emotions and from clearly externally focused emotions such as sympathy and empathy. The obtained correlations between different types of emotions also support this observation, and the function of regret also seems to be different than that of clearly internally or externally focused emotions.

The conclusions offered by our studies are limited in several respects. First and foremost, findings are based on correlational data therefore the described causal relationships are only hypothetical based on theoretical literature. As in all such studies, it is hard to establish to what extent participants actually experienced the emotions they reported and to what extent their responses were governed by their assumptions about what they *should feel* in the given situation. Another limitation is that while testing hypotheses in several different intergroup contexts has advantages on the one hand, the essential differences between these contexts raise important questions on the other hand. To what extent do today’s people identify with violent gendarmes who lived more than a century ago (and, to begin with, to what extent are gendarmes prototypical group members when the victimized out-group involved in the comparison is represented by university students of the same age as that of the majority of participants)? To what extent is the aggression committed by ice hockey fans comparable to that taking place between people living along the Hungarian–Serbian border? To what extent is it reasonable to expect participants to become involved in not particularly significant or widely known events which took place more than a century ago? For example, findings reported by Imhoff et al. (2013) suggest that Study 3 found weaker effects in general and no relationship between perceived in-group moral superiority and specific misdeed-related reparation in part because the events presented are already closed in a psychological sense, having completely been referred to the past. Moreover, these events may appear small compared to that construction of national history to which participants have been exposed since primary school. Pilecki and Hammack (2014) point out that the effect of events depicting the in-group’s aggression may be insignificant because by the time participants face such events, they have already been presented countless times with accounts suggesting the opposite view (i.e., that the in-group is the victim while the out-group involved in the event is the aggressor). Thus, the presented wrongdoing does not elicit reactions which would be constructive regarding intergroup relations. It is also somewhat problematic that perceived moral superiority was connected to different exonerating cognitions in different contexts. While this makes sense, it also makes the interpretation of our results less clear.

One of the most important limitations as well as an important factor to be considered in future research is that in 2010, when we began the present studies, we employed the construct of perceived in-group moral superiority in order to empirically assess collective victimhood of the Hungarians revealed by narrative psychological studies (László, 2013). We argued that viewing the in-group as a victim and the out-groups as aggressors may result in the perception of moral superiority of the in-group. Bar-Tal and his colleagues published a study on collective victimhood in 2009, and since then considerable efforts were made to develop instruments measuring this construct (e.g., Schori-Eyal et al., 2009; Vollhardt and Bilali, 2015; Vollhardt et al., 2016). In future research, the central hypotheses of our studies will require a specific measure of victimization experience instead of perceived moral in-group superiority since the latter is only an assumed consequence of the former.

In sum, the most important contribution of our studies to the literature is that they made an attempt to relate the general perception of the past (evaluating and comparing the in-group’s behavior with other groups’ behavior in a historical perspective) with the interpretation of events contradicting the perception of the in-group. The variable of perceived in-group moral superiority showed in the majority of cases a significant negative relationship with specific and general forms of reparation that provided further evidence of a dynamic relationship between the past and the present (Roccas et al., 2006; Wohl and Branscombe, 2008). Such interpretation of the past in relation to aggression led participants to either deny the aggression committed by the in-group, construe it as a mere reaction or judge it insignificant as compared to the out-group’s actions. Studying intergroup relations in the given region carries considerable importance since the Hungarians, Slovaks, Serbs, and other neighboring people have experience of several conflicts which still pose difficulties to the coexistence of these groups. Our studies attempted to empirically confirm the assumption that perceived in-group moral superiority as a particular way of constructing the past may pose an obstacle to maintaining harmonious intergroup relations.

## Author Contributions

ZS designed the studies, ZS, NM, and IC collected the data, ZS analyzed the data, ZS, NM, and IC interpreted the data. ZS drafted the article and NM and IC engaged in several rounds of critical revision of the article. ZS, NM, and IC contributed to the final approval of the version to be published.

## Conflict of Interest Statement

The authors declare that the research was conducted in the absence of any commercial or financial relationships that could be construed as a potential conflict of interest.
