# Corrigendum: The Role of Perceived In-group Moral Superiority in Reparative Intentions and Approach Motivation

**DOI:** 10.3389/fpsyg.2018.01442

**Published:** 2018-08-14

**Authors:** Zsolt P. Szabó, Noémi Z. Mészáros, István Csertő

**Affiliations:** ^1^Department of Social Psychology Eötvös Loránd University, Budapest, Hungary; ^2^Department of Social and Organizational Psychology University of Pécs, Pécs, Hungary; ^3^Department of Social and Organizational Psychology Pázmány Péter Catholic University, Budapest, Hungary; ^4^Research Center for Natural Sciences, Institute of Cognitive Neuroscience and Psychology Hungarian Academy of Sciences, Budapest, Hungary

**Keywords:** moral superiority, emotional attachment to the in-group, exonerating cognitions, group-based emotions, reparation

The following source of funding was not reported in the original publication: National Research, Development and Innovation Office – NKFIH, Grant Number K 109009. The authors apologize for this error and declare that the amendment does not affect the scientific conclusions of the published studies in any way.

The original article has been updated.

## Conflict of interest statement

The authors declare that the research was conducted in the absence of any commercial or financial relationships that could be construed as a potential conflict of interest.

