# Supplementary material for: The Role of Perceived In-group Moral Superiority in Reparative Intentions and Approach Motivation
Source: Front Psychol. 2017 May 31;8:912. doi: 10.3389/fpsyg.2017.00912 (PMC5450564; doi:10.3389/fpsyg.2017.00912)
Supplement: Supplementary file 1 [file Data_Sheet_1.docx]

Appendix A, B, C, D

Appendix A:

Anti-Serb inciting graffiti proliferate again in the border region

Release date: 12:58 12.05.2007

In the last few months, increasing amounts of Anti-Serb graffiti have appeared again in several towns in the border region. At the end of February, graffiti inciting extermination of the Serbs were left on several buildings in Subotica. The brick walls of the Serbian church bore the message „Chetniks die, we spread fear” painted by an unknown offender, while other offenders wrote the slogan „10 Serbs for 1 Vojvodina Hungarian!” on the entrance of the local sports ground.

The provocation in Subotica is far from being the only incident in the border region. A few months earlier, on the eve of 17 March, rioters targeted Senta where they painted swastikas and messages inciting against the Serbian community on several public buildings and private houses, they smashed shop windows and raided a pub. In the Hungarian town of Szentendre, the Serbian church was damaged by unknown offenders.

During the same night, a similar incident occurred in Szeged. This time the message „srpski pod noz”, that is, „Serbs under the knife” was painted on the wall of a private house.

A few days prior to the March incidents, Senta mayor Attila Juhász declared that the number of ethnically motivated criminal offenses had reduced in the region.

Despite the statement, many are afraid that the 2004 and 2005 events will be repeated. It is well known that in those years Anti-Serb atrocities were committed almost on a daily basis. Then both the Washington Congress and the European Parliament issued a statement condemning the intimidating acts.

In early May this year, the public was provoked by Anti-Serb graffiti left on the walls of the Petőfi Sándor Primary School in Deszk. The message read „Vojvodina belongs and will belong to the Hungarians!” Deszk mayor Péter Knézi issued a statement condemning the act of vandalism and declared that the incident was a single, exceptional case. This time the offenders were found soon after the incident due to the prompt and efficient reaction of the Hungarian police.

Appendix B:

Ice Hockey World Championship: The International Ice Hockey Federation warned the Hungarians again.

The 73rd Ice Hockey World Championship has begun in the country of cantons, in which Hungary has had the chance to participate once again after seventy years. Thousands of Hungarian supporters have arrived at Zurich to encourage their favourites during the Championship. There is a comparably large number of Slovakian supporters as well. However, the events having taken place during the Hungary vs. Slovakia game might be regarded anything but the celebration of sports. The game ending by a 4-3 Slovak win at the last moment was disturbed by the tumultuous Hungarian supporters from the beginning. Firecrackers were first thrown to the rink from the „Hungarian” sector, then the Hungarian fans began chanting „You have no homeland! Who is not jumping is a Slovak, hey, hey!” After the game was finished, the Hungarian fans jeered off the Slovakian national anthem honouring the winning team, and then they turned their back on the team. Subsequently, Hungarian fans hit and kicked a Slovakian supporter. Since such disturbance has so far been unprecedented at ice hockey world championships, it is not a surprise that the general secretary of the Hungarian Ice Hockey Federation Zoltán Kovács expressly condemned the incidents. The international federation has held out the prospect of punitive sanctions.

Appendix C:

The Černová shooting

103 years ago in a Slovakian village in Liptó county, 15 people were shot dead by Hungarian gendarmes. In the early 1900s, local people built up a Catholic church encouraged by Andrej Hlinka, who was born in the village. The church was finished soon – the village was arranging for consecration as early as 1907. Locals wanted Hlinka to consecrate the church, but he had previously infringed the law banning incitement against the Hungarian nation during the 1906 electoral campaigns, so he was not allowed to participate in the ceremony. Nevertheless, locals insisted that Hlinka should conduct the consecration. As a result of threatening letters, the bishop first and then his deputy also resigned from conducting the ceremony. In the meantime, Hlinka relinquished his privilege in a letter. Márton Pazurik, the churchman who eventually undertook the function went to the village with the letter to appease locals. However, they were so much excited that a detachment of gendarmes had to be posted in Černová. At the same time, two coaches arrived, one bringing higher churchmen including Pazurik, the other occupied by the district administrator and an official attendant. By that time, hundreds of locals had blocked the road at the village gate in order to prevent the consecration. The coaches, albeit guarded only by seven gendarmes, drove into the crowd, which then attacked them, and the Hungarian gendarmes opened fire in response. Nine died at the scene, six more died during subsequent days, and several people were wounded severely or lightly.

Appendix D:

The Nationalities Law passed following the Austro-Hungarian Compromise of 1867 was almost unprecedented at the time of enactment. However, it was received rather unfavourably by the Hungarian public and political leaders as well as by the nationalities. In the Hungarians’ view, the rights granted by the law were too generous while the nationalities were dissatisfied with their narrow scope. From the 1870s, the regulations became increasingly strict as a result of repeated reviews. At the end of this process, the Hungarian national assembly passed an act on linguistic rights that prescribed mandatory use of the Hungarian language in the entire territory of Hungary. The act was not accepted without difficulties by the Croatian nationality, which strove to establish equality-based partnership relations with the Hungarian majority. Hungarian-language notices were placed in the streets and public buildings. Zagreb university students protested against the act. The protests were broken up by the Hungarian gendarmerie, which severely wounded several protesters.
